# Supplementary material for: Simulation modeling to assess performance of integrated healthcare systems: Literature review to characterize the field and visual aid to guide model selection
Source: PLoS One. 2021 Jul 9;16(7):e0254334. doi: 10.1371/journal.pone.0254334 (PMC8270171; doi:10.1371/journal.pone.0254334)
Supplement: S1 Table — (DOCX) [file pone.0254334.s003.docx]

**S1. Search terms for systematic search.**

| Techniques **terms** | IC Domains **terms** | Health **term** |
| --- | --- | --- |
| "Agent-Based" OR "Complex systems" OR "Complexity science" OR "Computer model" OR "Computer simulation" OR "Discrete event" OR "Heuristics" OR "Integer programming" OR "Linear programming" OR "Markov" OR "Mathematical model" OR "Mathematical optimization" OR "Mathematical programming" OR "Metaheuristics" OR "Modelling patient" OR "Monte Carlo" OR "Network analysis" OR "Operational management" OR "Operational research" OR "Operations management" OR "Operations research" OR "Optimisation" OR "Optimization" OR "Organizational model" OR "Queueing" OR "Queuing" OR "*Simulation" OR "Statistical model" OR "Stochastic analysis" OR "Stochastic model" OR "Stochastic modelling" OR "Stochastic process" OR "Systems approach" OR "Systems model" OR "System dynamics" OR "Systems theory" OR "Systems thinking" OR "Systems Science" OR "Theoretical Computer model" OR "Visual simulation" | “integrated care” OR "care access" OR "adequate care" OR "responsiveness" OR "appropriate care" OR "coordination" OR "amenable mortality" OR "preventable death" OR "avoidable death" OR "balance of care" OR "care balance" OR "care planning" OR "planed care" OR "transitions" OR "carer experience" OR "provider experience" OR "case management" OR "chronic disease management" OR "chronic disease" OR "long term" OR "integration" OR "communication" OR "community" OR "continuous care" OR "continuity of care" OR "care continuity" OR "care continuum" OR "cost" OR "decision support" OR "shared decision" OR "diabetes" OR "disease management" OR "emergenc*" OR "experience of care" OR "care experience" OR "patient experience" OR "user experience" OR "financial protection" OR "health" OR "health care home" OR "home care" OR "care home" OR "health improvement" OR "health increase" OR "better health" OR "improve health" OR "health it" OR "health analytics" OR "health data analytics" OR "HIT" OR "health data management" OR "healthcare data management" OR "health outcomes" OR "health results" OR "lifestyle" OR "hospital admissions" OR "readmissions" OR "hospital use" OR "hospital utilisation" OR "hospital resources" OR "independent living" OR "information" OR "interpersonal" OR "medication management" OR "organisational processes" OR "system structure" OR "patient centred" OR "patient safety" OR "population health" OR "population needs" OR "prevention" OR "public health" OR "self-management" OR "self" OR "service improvement" OR "better service" | “Health*” |
