# Supplementary material for: Simulation modeling to assess performance of integrated healthcare systems: Literature review to characterize the field and visual aid to guide model selection
Source: PLoS One. 2021 Jul 9;16(7):e0254334. doi: 10.1371/journal.pone.0254334 (PMC8270171; doi:10.1371/journal.pone.0254334)
Supplement: S2 Table — (DOCX) [file pone.0254334.s004.docx]

**S2. List of simulation modeling techniques and integrated care topics-of-interest used in selection criteria**

| **Simulation Modeling techniques** | **ICS-interest areas** | |
| --- | --- | --- |
| Discrete event simulation  Monte Carlo simulation  System dynamics  Agent based models  Hybrid simulation  Markov Model  Cohort Simulation  Micro Simulation  Gaming simulation  Inverse simulation  Real time simulation  Other- Self identified as simulation model | Amenable mortality  Healthy lifestyles  Population health  Hospital admissions  Hospital readmissions  Community-based care  Specialist care \| Diagnostics  Patient safety  Health outcomes  Quality of life  Independent living  Self-management  Patient experiences of care  Experiences  Continuity of care  Supporting holistic goals and outcome  Communication and information  Shared decision making  Care planning | Care delivery and transitions  Emergencies  Use of care services  Hospital utilization  Residential and long-term care utilization  Primary care utilization  Health care costs  Balance of care  Advancement of integration  Access to care  Hospital use  Care transitions  Medications management  Care coordination  Care quality  Staff/ workforce  Case management*  Establish accountability or negotiate responsibility. |
