# Supplementary material for: Simulation modeling to assess performance of integrated healthcare systems: Literature review to characterize the field and visual aid to guide model selection
Source: PLoS One. 2021 Jul 9;16(7):e0254334. doi: 10.1371/journal.pone.0254334 (PMC8270171; doi:10.1371/journal.pone.0254334)
Supplement: S3 Table — (DOCX) [file pone.0254334.s005.docx]

**S3. Data extraction sheet**

| **Data** | **Explanation** | **Instruction** |
| --- | --- | --- |
| **Reference:** |  |  |
| Study name | Study name | Text |
| Author | Main Author name | Text |
| Journal | Journal name | Text |
| Year of Publication | Year of publication | Text |
| Country | Country of the assessment | Text |
| Funding source | Funding source | Text |
| Key concept | Short description of the article topic | Text |
| **Techniques:** |  |  |
| Primary simulation method | Simulation method used in the article | Method name |
| Secondary method | Secondary method used in the article | Method name |
| Other Methods | Others methodologies used in the article | Method name |
| Analytical Perspective | What is the perspective adopted for the evaluation (health service, third-party payer, societal?) | Text |
| Purpose | The purpose of the model. | Text |
| Implementation level | At what level of the healthcare system is the simulation implemented | Healthcare system level |
| Strengths | Documented strengths of the model | Text |
| Limitations | Documented limitations of the model | Text |
| **Problematic:** |  |  |
| Research Question | Research question/objectives if question not stated | Text |
| IC Level | ICPA framework level of the article | list |
| IC Dimension | ICPA framework dimension of the article | list |
| IC type of Indicators | ICPA framework type of indicators related to either inputs or outputs of the model | Text |
| Necessity | Explanation of the problematic | Text |
| Population | Description of the population in the simulation | Text |
| Components (variables) in the model | All variables included in the simulation model. If graphic picture of the model is self-explicative-must be included. | Organized text; list |
| Interventions | Short description of the interventions | Text |
| **Resources:** |  |  |
| Time | Time required for full assessment | Days |
| People | People involved in the full assessment | Number |
| Type of data required (inputs) | Classification of the type of inputs needed for the model | Organized text; list |
| Data Required (inputs) | Specifications about the data used in the analysis | Text |
| Data Sources | Data sources | Text |
| Software | Software used in the Analysis | Text |
| **Results:** |  |  |
| Time horizon | Time horizon of the results | Years |
| Outputs | Measures resulting from the assessment | Organized text; list |
| Conclusions | Article main conclusions | Text |
| Implications | Article implications in real implementation / policy | Text |
| Reviewer Critique | Reviewer Critique | Text |
