# Supplementary material for: Simulation modeling to assess performance of integrated healthcare systems: Literature review to characterize the field and visual aid to guide model selection
Source: PLoS One. 2021 Jul 9;16(7):e0254334. doi: 10.1371/journal.pone.0254334 (PMC8270171; doi:10.1371/journal.pone.0254334)
Supplement: Attachment — Submitted filename: Response to Reviewers.docx [file pone.0254334.s006.docx]

Nicolas Larrain, Ph.D. (Candidate)

Hamburg Centre for Health Economics, University of Hamburg. OptiMedis AG

Burchardstraße 17; 22303. Hamburg, Germany.

19.11.2020

Dear reviewers and Academic Editor;

We deeply appreciate your constructive critique and valuable comments. We have extensively revised the manuscript to address each of the concerns. We have taken the liberty of organizing your comments in the following table, where the first column shows the revisions by each reviewer and Academic Editor, and the second column presents our answers explaining how we have addressed each revision.

| **Revision** | **Answer** |
| --- | --- |
| Reviewer 1: | |
| Authors introduce the topics very clearly, exploring the whole health care performance context thoroughly. They illustrate the issue the paper should contribute to address: how to keep into account complex interactions amid factors those which impact triple aim. They also specify that their review wants to explore possible simulations for improving “what if and “how to” scenario processes, listing the major limits of actual models proposed by some institutions. Final aim – presenting a visual aid to select the most appropriate simulation - is clearly expressed.  Methods section details search strategy. Inclusion ad exclusion criteria are clean.  Authors discuss data extraction and analysis which they adopted. They carefully show the process they followed, focusing how each step contributes to an integrated frame which looks coherent with paper’s aims. | Thank you very much for your positive assessment of our methodological approach. |
| Results  The description of Areas of Assessment is readable as it connects its themes with the topics in a structured way. The PRISMA diagram contributes to clarity.  Table 1 classifies selected papers by described criteria.  The description of simulation modelling techniques starts classifying them by five categories and adding a sixth one which includes studies based on three or more models (hybrid). The section structure is coherent but it could have started explaining reasons behind authors’ choice. Even though it should be considered a minor issue, it would add value trying to adopt a specific classification for simulation models. In case criteria available do not fit due to specificity of authors aims, it could help to quickly explore the connection between this classification and those are often adopted in decision making under uncertainty (e.g see the book Kochenderfer, M.J., 2015, Decision Making Under Uncertainty (MIT Lincoln Laboratory Series) The MIT Press.). | The reviewer is correct in raising that the classification used for Simulation models and the structure used in the results section requires further explanation. As the reviewer mentioned, the reason is that criteria previously reported in the literature do not fit well the practical applications of our study, which we have now further specified. Furthermore, several reviews have been published with different approaches of classifying simulation models. Our structure choice purposely differentiates from these classifications in an effort to expose clearly the added value and practical implication of our paper. Nevertheless, we do agree that an explanation of our choice was necessary and was added in section 2.4, on lines 138 - 155, including the references that guided each item.  We thank the reviewer for the reference provided. In studying it we believe that its scope is more aligned with classifications responding to technical and mathematical issues (presented in a complexity progression typical of academic books) more than an exposé of usefulness for a particular area(Integrated care), like our work. We have however added a short mention to decision making under uncertainty on the Discussion section, lines 431-437 of the final manuscript. |
| The table offers a synthetic view which allows readers getting the big picture. Once again, as minor issue authors should have quoted the sources they based upon to state strengths and limitations of different types of models. It could be especially helpful for readers who do not have specific background. | We thank the reviewer and agree with the assessment The sources have been referenced in the table. |
| The Complexity section is especially interesting and at the center of authors aims. Lack of complexity in evaluating health care performances is one of the issues that authors want to address, so this section is expected to be rigorous and original. The latter expectation is quite satisfied while it is not possible to evaluate the first. Authors list nine complexity features those which are present in the 27 simulation models they’d previously selected. These features sound impactful and relevant but author should quote studies to help readers see them in the broader frame of system modelling theories. | We thank the reviewer for the positive assessment of the originality of our study. In order to allow evaluation of the rigor employed, we have now added an expanded explanation of the common framework to evaluate complex relations between system components (section Results; 3.3 Complexity; lines 333-346). References for the terms and concepts in the different fields were provided. |
| It also should allow to better understand both the connection among these features and how they impact on estimating health care system performance. | We agree and have updated the explanations for better internalization of the concepts (lines 347-391). |
| Some features look ambiguous if considered outside a frame of reference as in different field they are referred to different phenomena. E.g. dynamism in dynamic system theory could refer to different meanings ranging from the presence of state variables to the time-variant characteristic of the system itself or both. Again, authors connect Adaptation with intelligence as the ability to make decisions following specific rules. While it is a possible option, the definition of dynamism few rows above could bring someone wonder whether these rules must vary over time for a model showing dynamism. | We thank the reviewer for raising this issue. We added references to a common framework to consolidate the concepts from different fields. (lines 333-391) |
| In summary, while this section is relevant and innovative, authors should better explain references and help readers put these features in a unitary frame. | We are particularly thankful for this comment as it motivated us to present and give central importance to the complexity framework we had developed. |
| **Discussion**  The discussion starts exploring why a family of models can be helpful in modelling a specific system. While the intent is correct, this part sounds a little bit narrative. Maybe a more schematic description could help to stay connected with both the nine features and the five types of models described above. | Based on the reviewer´s comment we have now reorganized this section and changed the discussion with two main focuses. 1^st^ to discuss the advantages for integrated care of implementing systems thinking, including a summary of the contribution of each modeling technique and the discipline in general in this regard.  2^nd^ On the usefulness of our research and how to use the tool that summarizes our findings. |
| This part looks as it was written through some and partial examples, so the reader could wonder why other considerations were neglected by authors and especially why. |  |
| In the second and in the third part authors discuss the core of the paper: how to improve the choice of performance simulation model for evaluating health care performance in the late of the “triple aim”.  They begin explaining how different models can cope with different health care systems, then they explain how they applied these concepts to design the visual tools, showed in fig. 2.  The visual tools is exciting for its simplicity. Authors illustrate through one example how to apply it for choosing a simulation model which fits both your needs and constraints. Nonetheless it shows a major weakness: authors do not specify how to integrate its different components. E.g. if the system under scrutiny calls for more than one relevant feature should the user follow different connectors, probably ending up in more than one loop? In this case, should users integrate different models? | As we said before, we thank the reviewer for pushing us to give more emphasis to our selection tool. We have added a general rationale for use of the tool (Discussion, lines 451-456) and a third example that covers how to deal with a system with multiple parallel complexities (467-479). The key to the use of the selection tool is the identification and prioritization of the complexities that rule the system’s performance.    We acknowledge the limitation of the tool to integrate several complexities with a different priority. However, after the selection of the most important complexity to be modeled, the transit options will integrate the most common second or third level complexities associated with systems ruled by the selected top complexity. We believe the practical utility of our tool to orient users towards an appropriate modeling approach justifies our approach. |
| Authors should be more systematic discussing the tool which should be considered like a model itself, as it offers a way to choose simulation models through matching the features proposed by authors, as a result of their review, and needs coming from triple aim approach. More examples should be proposed to help readers understand how to use the tool when more than one feature is necessary. |  |
| **Limitations** are expressed clearly. |  |
| **Conclusion** suffers the weakness posed in the discussion section. | Based on the reviewer's comments, we have updated our conclusion in line with the new structure of the discussion. |
| **Summary**  Positive  • Relevant goals and clear expression of the issues to address  • Impactful contents  • High quality review process  • Clear language  To improve  • Clarify classification criteria  • Better connect the nine features in a common frame  • Explore and explain how to use the visual tool when health system calls for multiple features and needs. |  |
| Reviewer 2: | |
| While this paper is well written, and discusses a timely topic that in principle is worthy of academic investigation, I do not think that the paper should be accepted for publication. In my view, the paper provides an interesting overview of recent simulation modeling efforts in health systems (with a special emphasis on complexity, being an important characteristic of such systems). But it does not go beyond that, and indeed more is needed to justify publication in a scholarly journal. | We thank the reviewer for stating that we are providing an interesting overview of recent simulation modeling efforts in health systems, one of the objectives of our paper.  However, we kindly disagree that our paper does not go beyond that.  First, while we clearly state that previous reviews have been conducted in the field, some of them are quite outdated. Our review provides a thorough and up-to-date overview of current approaches in the field.  Secondly, as also pointed out by reviewer one we have presented an innovative approach to help users identify the most appropriate simulation technique, given a set of criteria. We believe that our presentation of this approach would indeed stimulate scholarly debate and may lead to further refinements of the method. |
| First, the paper misses a scientific motivation. The justification for the paper is given in lines 70-72 and can be summarized by saying that simulation models have recently gained more recognition. But this in itself is no (scholarly) motivation for reviewing the related literature. A proper motivation would include: a discussion of why and to whom such a review would be beneficial, i.e., what is the aim of doing such a review? Who would use it, and to what aim? What goes wrong when the study (review) is not done? | We thank the reviewer for the reflection which we considered carefully. The assessment of the reviewer might be informed by the expectation of a typical PICO style presentation of the research questions, which however does not apply to our study subject. In order to respond to the points raised by the reviewer, we have re-written the Introduction to better state the scientific motivation(57-59), the knowledge gap(71-82.), our aim(83-86) and objectives and the audience of the review(86-88).  In short, the paper’s motivation relates to the lack of tools to assess integrated care as a dominant health system reform strategy. The identified problem is that system thinking is missing in integrated care performance assessment framework.  A solution to this problem is simulation modeling. The identified knowledge gap is the link between the complex system perspective of SM and the usefulness for integrated care performance assessment. We close this gap by reviewing papers that use simulation models with systems thinking for evaluations in topics relevant to integrated care systems and extracting the particular features that are useful for evaluating integrated care. We focus on the features for implementation of system thinking in a common framework and culminate our results in a tool for guiding model selection. We hope that the reviewer is satisfied with these improvements to the manuscript. |
| Also, a scientific motivation would include the identification of a clear knowledge gap: what other review efforts have been published in the academic literature? What is missing in those reviews, making this review necessary? Can the health domain learn from reviews of simulation models in other fields? Especially in light of various recently published review papers (14, 24, 65) it is of paramount importance that the authors justify the need for another review paper on this topic. Without such a clear motivation, the paper reads more like a (interesting) policy report, not a scholarly article. | We agree with the reviewer that an articulation of the knowledge gap required strengthening. We added a paragraph referencing previous literature and stating clearly the knowledge gap that we are covering with the review. Lines 71-82. |
| Second, although the paper provides an interesting overview of relevant papers and the properties of the models described in them, it does not offer as much as I hoped for, in terms of deep reflection and discussion of subtle connections between the models. This can be due to the fact that an enormously wide net is cast across a variety of modeling approaches, each of which having been studied in thousands and thousands of academic papers. Describing such canonical and grand modeling traditions in so few words inescapably leads to gross simplifications. Although understandable, this does not do justice to the subtleties and complexities of each of these models, let alone the nuanced relations between them. | We thank and acknowledge the limitation highlighted by the reviewer and have addressed it in the limitations section (lines 503-509).  Nevertheless, (as the added paragraph clarifies) we think that the provided simplifications don’t undermine the usefulness and academic value of our work. We acknowledge that an in-depth description of the identified models that would allow an analysis of the subtle connections between models would be impossible to undertake with our study design. Instead, we provide readers with an introduction to the identified simulation modeling techniques and highlight the characteristics that allow them to implement system thinking.  Furthermore, we provide a way forward by encouraging readers that find a solution in our work to the challenges they encounter when assessing the performance of a complex health system to learn in detail the technique that our paper has pointed towards. Such an approach, where an extensive literature/discipline is undertaken by a few practical applications to provide a practical way forward, is common in the academic literature and fundamental for bringing together different fields of expertise. An example is the paper by Behrendt & Groene 2016 (DOI: 10.1016/j.healthpol.2016.08.003) |
| Similarly, the applications of the discussed models in the healthcare domain are so diverse (see column IC Topic-of-interest in Table 1), that drawing commonalities and generic lessons from them is hardly possible. What can one learn from comparing the use of model A in context X with the use of model B in context Y? For this to lead to credible results, many more data points are needed. As a consequence, the paper results in a well-structured but unavoidably shallow discussion. | In agreement with the reviewer comment, we too believe that is not possible to discern clear patterns between models and types of application in integrated healthcare with such a small sample size. However, two incipient patterns do emerge; (1.) System Dynamics for “Policy and strategy” and (2.) Discrete Event Simulations for “Health Resource Management”. This analysis is mention in the Discussion (lines 419-431).  This being said, even though finding a relation between the type of model and the different applications would be useful for integrated healthcare managers, a similar classification has already been made (Jun et al 2011) and escapes our main objective, that is to characterize simulation modeling focusing on the ability to implement system thinking.  Instead, we provide the type of applications (section 3.1, line 168) as part of a comprehensive characterization of simulation modeling in integrated care, with the purpose of informing readers of the reach of the discipline in integrated care topics. The classification also serves the purpose of grouping complexities, and we think it will help readers to identify complexities in different settings. |
| These two issues together make that this reviewer, having read the paper, feels that the paper never really explained what its added value to the academic literature would be (point 1) and that it is indeed difficult to identify key lessons learned from the material that is presented (point 2). In combination, this makes the paper rather unsuitable for publication in a scholarly journal, although I can imagine that with some extensions the manuscript could be made suitable as a report for practitioners and policy makers, especially those with limited knowledge of simulation models and a wish to have a systematic, practical guide to help them navigate such models in the healthcare domain. | We thank the reviewer for the critique, which we have carefully considered and based on which we have substantially revised the manuscript.  With utmost respect, we provide a summarized answer to the two points raised by the reviewer.  Point 1: Exposing simulation models as a solution for the challenges of integrated healthcare systems performance assessment, focusing particularly on their ability to implement system thinking.  Point 2. A summary of key (to IHS) characteristics and complexity features in a common framework that helps in guiding the identification and prioritization of complexity, which in turn are useful to select an appropriate simulation model.  We strongly believe that our methodological approach adheres to rigorous standards and that our work goes far beyond the content expected for a policy brief or practical guide and indeed would inform scholarly debate. |
| Academic Editor | |
| 1. Please ensure that your manuscript meets PLOS ONE's style requirements, including those for file naming. The PLOS ONE style templates can be found at   - https://journals.plos.org/plosone/s/file?id=wjVg/PLOSOne_formatting_sample_main_body.pdf and - https://journals.plos.org/plosone/s/file?id=ba62/PLOSOne_formatting_sample_title_authors_affiliations.pdf | We thank the Academic Editor for your comments and guidelines. In response, we are pleased to declare the following:  We have formatted the manuscript as stated in the templates. |
| 2. Thank you for stating the following in the Competing Interests section:  "The authors have declared that no competing interests exist."  We note that one or more of the authors are employed by a commercial company: OptiMedis AG. |  |
| 2.1. Please provide an amended Funding Statement declaring this commercial affiliation, as well as a statement regarding the Role of Funders in your study. If the funding organization did not play a role in the study design, data collection and analysis, decision to publish, or preparation of the manuscript and only provided financial support in the form of authors' salaries and/or research materials, please review your statements relating to the author contributions, and ensure you have specifically and accurately indicated the role(s) that these authors had in your study. You can update author roles in the Author Contributions section of the online submission form. | We have provided an amended Funding Statement in the Cover letter as recommended |
| Please also include the following statement within your amended Funding Statement.  “The funder provided support in the form of salaries for authors [insert relevant initials], but did not have any additional role in the study design, data collection and analysis, decision to publish, or preparation of the manuscript. The specific roles of these authors are articulated in the ‘author contributions’ section.”  If your commercial affiliation did play a role in your study, please state and explain this role within your updated Funding Statement. |  |
| 2.2. Please also provide an updated Competing Interests Statement declaring this commercial affiliation along with any other relevant declarations relating to employment, consultancy, patents, products in development, or marketed products, etc. | We have provided an amended Competing Interests Statement in the Cover Letter as recommended. |
| Within your Competing Interests Statement, please confirm that this commercial affiliation does not alter your adherence to all PLOS ONE policies on sharing data and materials by including the following statement: "This does not alter our adherence to PLOS ONE policies on sharing data and materials.” (as detailed online in our guide for authors http://journals.plos.org/plosone/s/competing-interests) . If this adherence statement is not accurate and there are restrictions on sharing of data and/or materials, please state these. Please note that we cannot proceed with consideration of your article until this information has been declared.  Please include both an updated Funding Statement and Competing Interests Statement in your cover letter. We will change the online submission form on your behalf.  Please know it is PLOS ONE policy for corresponding authors to declare, on behalf of all authors, all potential competing interests for the purposes of transparency. PLOS defines a competing interest as anything that interferes with, or could reasonably be perceived as interfering with, the full and objective presentation, peer review, editorial decision-making, or publication of research or non-research articles submitted to one of the journals. Competing interests can be financial or non-financial, professional, or personal. Competing interests can arise in relationship to an organization or another person. Please follow this link to our website for more details on competing interests: http://journals.plos.org/plosone/s/competing-interests |  |
