# Supplementary material for: Simulation modeling to assess performance of integrated healthcare systems: Literature review to characterize the field and visual aid to guide model selection
Source: PLoS One. 2021 Jul 9;16(7):e0254334. doi: 10.1371/journal.pone.0254334 (PMC8270171; doi:10.1371/journal.pone.0254334)
Supplement: Attachment — Submitted filename: Response to Reviewers.docx [file pone.0254334.s008.docx]

Nicolas Larrain, Ph.D. (Candidate)

Hamburg Centre for Health Economics, University of Hamburg. OptiMedis AG

Oliver Groene, PhD MSc

Burchardstraße 17; 22303. Hamburg, Germany.

01.06.2020

Dear reviewer and Academic Editor.

We are deeply thankful for your revision. We have revised each of your comments and provide each with a response in the following table.

| **Editor Comments:** | |
| --- | --- |
| The revision has been reviewed by the reviewer from the last round. The reviewer has made a favorable recommendation.  I have gone through the revision again. I highly appreciate that the authors have seriously addressed some of the concerns. The scope of the work and research question are now clear.  I believe the work has certain degree of merit and potential to be published. However, since PLOS ONE publishes scientifically rigorous studies, there are still two major issues which have to be addressed: | We are deeply thankful to the Editor for recognizing our work in the article. We have made extensive changes to the core of the article to address the editors’ concerns. Regarding the Editor’s second comment, in summary, we understand that the current selection of articles is deemed to be more subjective than what is usually found in systematic literature reviews. In particular, because even though the criterion for selection is clearly defined, part of the definition (Section 2.2 line 155: <<Finally, we excluded from the data extraction and analysis studies whose reporting standards were insufficient to fully understand and replicate the assessment >>) leaves room for a subjective interpretation. While even in the most rigorous systematic reviews data extractors have to introduce a certain level of interpretation in deciding which study and which data to include, we agree that our approach previously has not been optimal. In order to correct for subjectivity, we have now included all the articles with the highest quality score following our quality assessment (+3 extra articles). Furthermore, to be transparent with reader expectations, we have decided to define our article as a literature review with systematic a search^[1]^. The PROSPERO record was modified stating this change. We have noted that PLOS ONE publishes all types of reviews of the literature (in terms of Grant and Booth typology), not only systematic reviews of the literature. However, by now labelling our study as literature review with systematic search we believe that our methodological approach meets all expectations a reader might have.  A detail of the implications of this change is given in the response to comment (2.) |
| 1. As compared with other similar studies on simulation modeling in healthcare system, the studies reviewed in this work (only 27) are significantly fewer. The arguments and conclusions made in this study are therefore not promising. This problem is particularly clear as shown in Section 3.2. For examples, the results presented are only based on 2 studies for Markov models, 1 study for microsimulation, 2 studies for agent-based models, 1 study for hybrid simulations, etc. What were claimed in those sections are not comprehensive and convincing. | We thank the editor for the comment. Even though we defend our article selection to be fit for purpose and aligned with the objective of the study, we recognize that the writing of section 3.2 leads readers to think that the revision of the different methodologies was based solely in the articles selected from the systematic search. This is not correct. The methodologies were extracted from the selected articles. Then, selected articles were used to explain each methodology features. Nevertheless, the features themselves were summarized using complementary literature (presented in table 2). This is explained in the methods section 2.4 (line 184-186). We have changed the first paragraph of the section 3.2 (lines 223-228) to better explain were the features explanation come from. The complementary literature used is not solely based on case articles, but mostly on review articles and books explaining the methodologies in detail. |
| 2. The problem stated in point 1 is probably caused by a subjective selection of articles (on p. 10) by only the two authors. This work is title a "systematic" review but this process systems to be non-systematic. I suggest there is a clear description of how the reviewers determine whether to include a study is included. A quality score is given to an article by the two authors; however, is this quality score reliable? (Do the authors imply that out of 2271 articles, only 27 are of quality? Others are not of quality?) Another issue is that a systematic literature review is to identify trends in the research area. It would be necessary to include studies not only based on the subjectively determined quality but need to identify the overall trends. | First, please see above regarding the change in the title of the document according to Grant´s and Booth’s typology.  Second, we edited the methods section, stating explicitly that articles were selected according to our appreciation of their reporting standards to allow for the objective of our paper(section 2.2). In other words, the final selection criteria relate to the ability of reviewers to fully understand and replicate the article for a complete assessment of how the complexities of the system were included in the model. The limitations section was also edited stating the limitation of the selection process.  Three extra articles were added following the correction in the selection process:   1. Ansah et al. 2016: The article was initially excluded because even though evaluated with quality A, it did not add any additional information to the analysis. Following the corrected exclusion criteria, the article is now added in the review. 2. Goldman et al. 2004: The article was not included in the original revision because even though the model has top quality, the practical application and validation of said model are found in subsequent articles. Meanwhile, articles depicting practical applications of the model lacked a detailed description of the model. Initially, we though evaluating them together as a whole was a reach of our quality assessment criteria, hence the initial exclusion. 3. Comans et al. 2017: Similarly, to number (1.), the paper was excluded because it did not add information for analysis. Following the corrected exclusion criteria, the article is now added in the review.   We included the assessment of the new articles and mention them as examples to explain system thinking where they contributed to the analysis. Nevertheless, the results, discussion and conclusion did not change (with exception of the count of optimization capabilities and time frame for assessment) with the inclusion with the new articles.  The abstract was edited showing these changes.  We understand that many systematic reviews indicate research trends, but we are certain that research trends are only reported when in line with the objectives of the study. Our article did not focus on pooled effect sizes or research trends, as the explanation of how SM methods are able to integrate system thinking is not time dependent. In the same line, the selection of a SM method is determined by identifying complexities and the overall objective and not on trends.  Furthermore, given that we have chosen to classify the article as a literature review (where reporting research trends is less common), we think that reporting research trends is less justified. |
| **Reviewers' comments:** | |
| 1. If the authors have adequately addressed your comments raised in a previous round of review and you feel that this manuscript is now acceptable for publication, you may indicate that here to bypass the “Comments to the Author” section, enter your conflict of interest statement in the “Confidential to Editor” section, and submit your "Accept" recommendation.  Reviewer #1: All comments have been addressed | We thank the reviewer for his positive appreciation. |
| 2. Is the manuscript technically sound, and do the data support the conclusions?  The manuscript must describe a technically sound piece of scientific research with data that supports the conclusions. Experiments must have been conducted rigorously, with appropriate controls, replication, and sample sizes. The conclusions must be drawn appropriately based on the data presented.  Reviewer #1: Yes | We thank the reviewer for his positive appreciation. |
| 3. Has the statistical analysis been performed appropriately and rigorously?  Reviewer #1: N/A | - |
| 4. Have the authors made all data underlying the findings in their manuscript fully available?  The PLOS Data policy requires authors to make all data underlying the findings described in their manuscript fully available without restriction, with rare exception (please refer to the Data Availability Statement in the manuscript PDF file). The data should be provided as part of the manuscript or its supporting information or deposited to a public repository. For example, in addition to summary statistics, the data points behind means, medians and variance measures should be available. If there are restrictions on publicly sharing data—e.g. participant privacy or use of data from a third party—those must be specified.  Reviewer #1: Yes | We thank the reviewer for his positive appreciation. |
| 5. Is the manuscript presented in an intelligible fashion and written in standard English?  PLOS ONE does not copyedit accepted manuscripts, so the language in submitted articles must be clear, correct, and unambiguous. Any typographical or grammatical errors should be corrected at revision, so please note any specific errors here.  Reviewer #1: Yes | We thank the reviewer for his positive appreciation. |
| 6. Review Comments to the Author  Reviewer #1: Is the manuscript technically sound, and do the data support the conclusions?  The authors have progressively addressed the original version issues. Their paper now sounds informative while it illustrates its aims and methods sharply. The authors illustrate their framework clearly. Now the flow is logical, and connections between scope, data, and conclusions are evident.  Have the authors made all data underlying the findings in their manuscript fully available?  Yes, they did.  Is the manuscript presented in an intelligible fashion and written in standard English?  The authors have progressively evolved their manuscript through discourse with the reviewers. After the last revision, the paper will offer a novel and informative perspective to health care scholars and especially professionals. It will help readers look at the “Triple Aim” model with a broader framework to assess health care performances considering complexity accurately and robustly. Though the proposed tool has limits, it shows the readers the relevance of the systemic approach in evaluating a health care system. The paper starts a practical conversation that goes beyond simple improvement in health care assessment techniques. This proposal falls in the field of accurate and relevant feedback conversation (e.g, see Zollo, 2009 about superstitious learning). Despite its limits, the tool connects the diverse elements that concur in performance and allows H.C. professionals to take them and their complex interactions into account in evaluating and comparing performance.  This reviewer can't judge the English level of the manuscprit. | We thank the reviewer for the recognition of our work, and we appreciate his positive appreciation and useful comments during the revision process. |

1. As defined by Grant & Booth. Grant MJ, Booth A. A typology of reviews: an analysis of 14 review types and associated methodologies. Health Info Libr J. 2009 Jun;26(2):91-108. doi: 10.1111/j.1471-1842.2009.00848.x. Review. PubMed PMID: 19490148
